# To leave no one behind: Assessing utilization of maternal newborn and child health services by all the 13 particularly vulnerable tribal groups (PVTGs) of Odisha, India

**DOI:** 10.1186/s12961-023-01101-7

**Published:** 2024-01-22

**Authors:** Jyoti Ghosal, Madhusmita Bal, Arundhuti Das, Bhuputra Panda, Manoranjan Ranjit‬, Manas Ranjan Behera, Sonali Kar, Sudhir Kumar Satpathy, Ambarish Dutta, Sanghamitra Pati

**Affiliations:** 1 School of Public Health, KIIT Deemed to Be University, Bhubaneswar, Odisha India; 2 ICMR-Regional Medical Research Center, Bhubaneswar, Odisha India; 3 Kalinga Institute of Medical Sciences, KIIT Deemed to Be University, Bhubaneswar, Odisha India; 4 Indian Institute of Public Health, Public Health foundation of India, Bhubaneswar, Odisha India

**Keywords:** Particularly vulnerable tribal groups, PVTGs, Odisha, Utilization, Maternal health, Newborn health, Child health, India

## Abstract

**Background:**

Indigenous tribal people experience lower coverage of maternal, newborn and child healthcare (MNCH) services worldwide, including in India. Meanwhile, Indian tribal people comprise a special sub-population who are even more isolated, marginalized and underserved, designated as particularly vulnerable tribal groups (PVTGs). However, there is an extreme paucity of evidence on how this most vulnerable sub-population utilizes health services. Therefore, we aimed to estimate MNCH service utilization by all the 13 PVTGs of the eastern Indian state of Odisha and compare that with state and national rates.

**Methods:**

A total of 1186 eligible mothers who gave birth to a live child in last 5 years, were interviewed using a validated questionnaire. The weighted MNCH service utilization rates were estimated for antenatal care (ANC), intranatal care (INC), postnatal care (PNC) and immunization (for 12–23-month-old children). The same rates were estimated for state (*n* = 7144) and nationally representative samples (*n* = 176 843) from National Family Health Survey-5.

**Results:**

The ANC service utilization among PVTGs were considerably higher than national average except for early pregnancy registration (PVTGs 67% versus national 79.9%), and 5 ANC components (80.8% versus 82.3%). However, their institutional delivery rates (77.9%) were lower than averages for Odisha (93.1%) and India (90.1%). The PNC and immunization rates were substantially higher than the national averages. Furthermore, the main reasons behind greater home delivery in the PVTGs were accessibility issues (29.9%) and cultural barriers (23.1%).

**Conclusion:**

Ours was the first study of MNCH service utilization by PVTGs of an Indian state. It is very pleasantly surprising to note that the most vulnerable subpopulation of India, the PVTGs, have achieved comparable or often greater utilization rates than the national average, which may be attributable to overall significantly better performance by the Odisha state. However, PVTGs have underperformed in terms of timely pregnancy registration and institutional delivery, which should be urgently addressed.

**Supplementary Information:**

The online version contains supplementary material available at 10.1186/s12961-023-01101-7.

## Introduction

Despite strides made to ameliorate maternal newborn and child health (MNCH) and their well-being as key components of sustainable human development, still in 2020, almost 800 mothers died worldwide each day from preventable causes related to pregnancy and childbirth [1, 2]. India continues to face challenges, even after considerable decline in its maternal mortality ratio (MMR); from 212 deaths per 100 000 live births in 2007 it stands at 97 per 100 000 in 2018–2020 [3], whereas the corresponding rate in the Organization for Economic Cooperation and Development (OECD) countries—a forum of high-income countries—stands at 22 deaths per 100 000 live births in 2020 [4]. Similarly, there has been considerable reduction in under 5 years child mortality rates (U5MR) also, from 64 deaths per 1000 live births in 2009 to 32 deaths in 2020 [5], the corresponding figures for OECD countries being 6 deaths per 1000 live births in the year 2021 [6].

However, despite declines in maternal and under 5 years old (U5) mortality in recent years, certain groups in India are systematically worse off than others especially with regards to these key indicators [4, 6–8]. Indian tribal people comprise such a group, who are considered a marginalized key population of India from multiple developmental dimensions [9]. Although the tribal population of India have shown a decline in U5MR rates over the years [95.7 deaths per 1000 live births (2005–2006) to 50.3 deaths (2019–2021)], still their U5MR is more than one and a half times that of their non-tribal counterparts [10].

India is the abode of 705 tribal communities, which account for about 8.6% of national population [11]. These populations are designated as scheduled tribes (ST) as per constitution of India [11]. The tribal population of India subsumes a few special tribal groups known as particularly vulnerable tribal groups (PVTGs) [12]. PVTGs, the most vulnerable subpopulation of India, (1) reside in hard-to-reach areas, (2) have pre-agricultural level of technology and practice subsistence level economy, (3) are experiencing a dwindling or stagnant population and (4) pervasively have an extremely low literacy [12]. A total of 75 PVTGs have been recognized in India, of which the state of Odisha is host to maximum number of PVTGs (*n* = 13), followed by 12 in Andhra Pradesh and 9 in Bihar including Jharkhand [12, 13].

As mentioned above, the tribal pregnant women and their children often face greater risk during and after pregnancy, leading to higher rates of morbidity and mortality [14]. Also, tribal people have been found to utilize the MNCH services that are implemented universally by the welfare state to prevent morbidity and maternity of mothers and children, at a significantly lower rate than the national average [14]. The PVTGs are likely to utilize even less because of their greater vulnerability due to geographical remoteness and social isolation. But there hardly exists any data on the MNCH service utilization indicators of PVTGs in India, because the national surveys do not identify them separately from the other tribal groups, and because of their small population they are unlikely to be represented in national samples adequately for any precise meaningful estimates. But it is well known that low service utilization are the major harbingers of poorer MNCH outcomes in vulnerable groups [15]. However, it is also well known that higher service utilization rates can lead to better and equitable MNCH outcomes despite the structural multi-dimensional deprivation any vulnerable group might be riddled with.

Therefore, estimating how this most vulnerable population in the country is being covered by the universal MNCH services would be very informative to the national as well global health policy-makers. After all, the fulfilment of the global promise of “leaving no one behind” hinges on how well the most vulnerable people are covered by key public health strategies. And given the fact that Odisha has one of the largest groups of PVTGs (tribal people constituting 22.85% and PVTGs constituting 0.7% of the state population as per the 2011 census) [16], it was perceived to be worthwhile to explore how the PVTGS of the state are performing in regards to MNCH services utilization-wise. Moreover, Odisha, a state with large tribal population among major Indian states, has been historically experiencing suboptimal health outcomes. Its tribal population has been worse off than the state average (selected tribal estimates have been presented in the Additional file 1: Table S1) [17]. This makes it even more relevant to study the most vulnerable tribal population of the state with regards to their MNCH service utilization—a critical step towards improved outcomes—for whom there hardly exists any information. The public health system of the state [18], which is often less than what is prescribed by Indian Public Health Standards, is described in the Additional file 1: Table S2 to provide insight to the context. Therefore, with this dearth of evidence in the backdrop, this study was envisaged to estimate the maternal newborn child healthcare (MNCH) service utilization by the PVTGs of Odisha, and, further, compare these utilization rates with that of the state and national rates. The study also aimed to compare the rates of caesarean section and the various dimensions of institutional delivery.

## Methods

An observational cross-sectional study on a representative sample of all the 13 PVTGs of Odisha was planned in Odisha, which is the abode of the highest number of PVTGs [13] in the country, followed by Andhra Pradesh (12 PVTGs—2nd highest). We studied a representative sample of all the 13 PVTGs of Odisha [ (1) Bonda, (2) Chuktia Bhunjia, (3) Didayi, (4) Dongria Kandha, (5) Hill Kharia/(6) Mankidia/(7) Birhor, (8) Juang, (9) Kutia Kandha, (10) Lanjia Saora, (11) Lodha, (12) Paudi Bhuyan, (13) Saora] between 5 November 2021 and 28 February 2023.

### Sample size estimation

An eligible household, the element of our sample, was one that had a mother and her most recently born child, born during the last 5 years preceding the survey and who was alive at the time of interview and living with their mother.

The size of a cross-sectional sample of households having eligible mother–child pair was calculated using the following formula:

$$n = \left[ {{\text{DEFF}}*{\text{Np}}\left( {{1} - p} \right)} \right]/[(d^{{2}} /Z^{{2}}_{{{1} - \alpha /{2}}} *\left( {N - {1}} \right) + \, p*\left( {{1} - p} \right)$$

*N* was the total PVTG population (*N* = 294 214). *p* was the expected prevalence of an outcome, which was estimated to be 10%. The rationale for selecting 10% as the expected prevalence stemmed from choice of 5% prevalence for outcomes by national (and internationally harmonized) surveys such as the National Family Health Survey (NFHS) and Longitudinal Ageing Study of India (LASI). As this study was part of a doctoral thesis, there was resource constraint; hence, the sample size was estimated with enough precision for outcomes with 10% prevalence, sacrificing some precision for rarer outcomes. Otherwise, the sample size would have been much larger and would have been beyond the resources of a doctoral study. *d* was the absolute precision, which was taken as 3%; the Z score corresponding to 95% confidence interval was 1.96; and the design effect was 3 to account for the clustered nature of the sample (see below in sampling technique). The sample size of households to be surveyed with the above parameters came out to be 1151.

### Sampling technique

The 13 PVTGs of Odisha live in villages, and those villages are clustered into micro-project areas (MPA) by the Odisha state administration for developmental purposes. There are 17 such MPAs, and they are of three types: (1) one PVTG per MPA (one-to-one; 5 such MPAs), (2) one PVTG spread over many MPAs (one-to-many; 11 such MPAs), (3) more than one PVTG in one MPA (many-to-one; 1 such MPA). The district-wise and MPA-wise list and population of PVTGs are provided in Table 1. A multi-stage sampling method was used to draw the study sample, which comprised the following stages:

**Table 1:** Detailed illustration of the population and sample distribution of 13 PVTGs, micro-project areas (MPAs)-wise and district-wise

Micro-project areas (MPA)	District	PVTG	Total population	Total village	Villages in the sample	Total households	Households in the sample
BDA, Mudulipada	Malkangiri	Bonda	16 357	75	6	3493	90
DDA, Kudumulugumma	Malkangiri	Didayi	10 854	50	10	2283	93
JDA, Gonashika	Keonjhar	Juang	38 853	250	7	8032	129
LDA, Moroda	Mayurbhanj	Lodha	8893	30	5	2064	88
CBDA, Sunabeda	Nuapada	Chutkia Bhunjia	4773	35	4	1147	66
DKDA, Chatikona	Rayagada	Dongria Kondha	12 772	85	6	2600	98
DKDA, Parsali	Rayagada				2		
LSDA, Serango	Gajapati	Lanjia Saora	48 713	218	10	10 697	151
LSDA, Puttasing*	Rayagada*						
SDA, Chandragiri	Gajapati	Saora	38 608	252	9	8178	120
TDA, Tumba*	Ganjam*						
KKDA, Lanjigarh	Kalahandi	Kutia Kondha	42 502	365	8	9324	132
KKDA, Belghar*	Kandhamal*						
PBDA, Rugudakudar	Deogarh	Paudi Bhuyan	67 870	270	11	15 263	159
PBDA, Khuntgaon*	Sundargarh*						
PBDA, Jamardhi*	Angul*						
HK&MDA, Jashipur	Mayurbhanj	Hill Kharia	4030	36	8	920	44
		Mankidia					16
		Birhor					

*These MPAs of respective districts were not covered

BDA, Bonda Developmental Agency; DDA, Didayi Developmental Agency; JDA, Juang Developmental Agency; LDA, Lodha Developmental Agency; CBDA, Chuktia Bhunjia Developmental Agency; DKDA, Dangaria Kandha Developmental Agency; LSDA, Lanjia Saora Developmental Agency; SDA, Saora Developmental Agency; TDA, Tumba Developmental Agency; KKDA, Kutia Kandha Developmental Agency; PBDA, Paudi Bhuyan Developmental Agency; HK&MDA, Hill-Kharia and Mankirdia Developmental Agency

Stage I: All five one-to-one MPAs and the only many-to-one MPA were automatically selected. Out of 11 one-to-many MPAs, 5 were randomly selected to cover all 13 PVTGs. An additional one-to-many MPA, DKDA, Parsali, was selected to get adequate Dongria Kondha households and members in the sample. So, a total of 12 out of 17 MPAs were included. Then the estimated sample of eligible households (*n* = 1151) was distributed to each MPA in such a way that the household/population selected for each PVTG roughly mirrored their proportion in the entire PVTG population. From smaller PVTGs enough numbers were chosen to have their adequate representation in the sample, although somewhat arbitrarily. However, their disproportionate allocation was adjusted using post-stratification estimation weights in the analysis stage. Then the number of households were divided by 20 to arrive at the number of villages to be sampled for each PVTG with a view that, on an average, 20 households will be selected from each sampled village.

Stage II: Villages were sampled using probability proportionate to size from the MPAs.

Stage III: 20 eligible households from selected PVTG villages were randomly selected. If 20 households were not found in one village, then nearby village(s) were linked to achieve the target.

### Study tools

A structured, valid, reliable, pretested and pilot-tested questionnaire was used for the study. The details of the tool development and validation procedure have already been published [19].

### Variables

The socio-demographic variables comprised districts, tribal groups, maternal age (categorized as 13–20 years, 21–30 years, 31–40 years, and 41–53 years), educational attainment in completed years (and then categorized as illiterate, 1–8, 9–10, 11–12 and above 12). Age at marriage and age at first pregnancy were converted into binary variables (< 18 years and ≥ 18 years). Gravida was summarized as mother having one, two, three and four or more live children born to them. The occupation and decision-making status of the mother within their family framework were also measured.

The antenatal care (ANC) consisted of 12 maternal, newborn and child health (MNCH)-related services such as pregnancy registration, early registration of pregnancy (within 3 months), mother and child protection (MCP) card received (Y/N), early ANC (within 3 months), four antenatal check-ups, five ANC components (measurement of weight, blood test, urine test, blood pressure, and abdominal examination), administration of two tetanus toxoid injections, iron tablets or syrup received and consumed for 100 or more days, mosquito net usage during the latest pregnancy, supplementary nutrition received (Y/N) and ANC counselling on institutional delivery, cord care, breastfeeding, kangaroo care, and family planning (Additional file 1: Table S3).

Intranatal care (INC) consisted of three indicators—institutional delivery (Y/N), skilled birth attendant (SBA)-assisted delivery (Y/N) and use of public ambulance service (Additional file 1: Table S3).

Postnatal care (PNC) also consisted of three indicators, mainly, timely PNC of mother and child (within 48 h after delivery), weight of baby after delivery (Y/N), and early initiation of breastfeeding (immediately after birth; Additional file 1: Table S3).

The immunization status comprised of all eight basic vaccinations, viz., bacillus Calmette-Guerin (BCG), Penta 3, Polio 3 and the first dose of measles received (Additional file 1: Table S3).

The place of delivery was further described as home, private and public. Similarly, place of immunization was categorized as private and public, and caesarean section was a binary variable (Y/N).

Mothers who delivered their child at home were asked the main reason behind doing so. This variable had a closed ended option.

### Comparator: National Family Heath Survey 5 (NFHS-5)

PVTG utilization rates were compared with that of Odisha state and national averages. Here, also, the mothers having their most recently born alive child 5 years preceding the survey were considered as a comparison groups. However, for assessing the status of all basic vaccination, the “very same group” was subcategorized into the child being aged 12–23 months for our sample as well as for two comparison groups.

### Statistical analysis

The socio-demographic variables of our study sample (*n* = 1186) were summarized using descriptive statistics. Proportions of PVTG mothers and their children undergoing ANC, INC, PNC and child immunization services were estimated. The estimation process used post-stratification probability weights to account for the slightly disproportionate representation of each PVTG in the sample as compared with their presence in the population. The PVTG estimates were then compared with corresponding service utilization estimates of Odisha state (*n* = 7141) and India (*n* = 176 843), estimated from NFHS-5 dataset using the usual survey weights of NFHS. The data analysis was carried out using R [20] and its Tidyverse [21] and *Arsenal* package [22].

### Ethical considerations

The study was approved by both the Institute Human Ethical Committee (ICMR-RMRC/IHEC-2020/12 dated 11/01/2020) and the Research & Ethics Committee of the Department of Health and Family Welfare, Government of Odisha (7033/MS-2-IV-04/2020(PT) dated 22/03/2021).

## Results

Our study sample (*n* = 1186) comprising all the 13 PVTGs of Odisha spanned across 86 villages from eight districts of the state. Paudi Bhuyan and Lanjia Saora were the most populous PVTGs and so had the largest representation in the sample, whereas Hill Kharia, Mankidia/Birhor were the least populated PVTGs, hence having the smallest representation in the sample. The representation of each PVTG in the sample approximately reflects their distribution in the state population (Table 1).

Mothers in the age group of 21–30 years made up 65% of the sample. However, only 11% and 2.5% mothers were below 21 years and above 40 years, respectively. Almost 58% of mothers were illiterate, and approximately one fourth of mothers attained a primary level of schooling. The predominant occupation was agriculture (76.4%). Almost 2 in 5 mothers got married before 18 years, and one in five experienced their first pregnancy before that age. Three fourths of the mothers had two or more pregnancies including the current one for which they were eligible to be included in the survey. Half of the mothers were involved in decision-making in their respective families (Table 2).

**Table 2:** Socio-demographic characteristics of PVTG mothers

Variables	Unweighted *n* (weighted %); *n* = 1186
Current age of mother (in years; *n* = 1186)	
13–20 years	144 (10.5%)
21–30 years	743 (64.6%)
31–40 years	269 (22.4%)
41–53 years	30 (2.5%)
Educational attainment (in completed years; *n* = 1186)	
Illiterate	708 (58.4%)
1–8	278 (22.4%)
9–10	156 (14.8%)
11–12	37 (3.7%)
Above 12	7 (0.7%)
Age at marriage (in years; *n* = 1186)	
Below 18 years	448 (36.1%)
18 years and above	738 (63.9%)
Age at primigravida (in years) (*n* = 1186)	
Below 18 years	255 (20.3%)
18 years and above	931 (79.7%)
Gravida (*n* = 1186)	
1	307 (28.3%)
2	350 (28.3%)
3	222 (19.0%)
4 or more	307 (24.4%)
Occupation (*n* = 1185)	
Agriculture	808 (76.4%)
Horticulture	4 (0.3%)
Daily wage	195 (10.7%)
Shifting cultivation	8 (0.3%)
Forest collection	81 (5.5%)
Food gathering	11 (1.3%)
Small business	5 (0.3%)
Fishing	1 (0.1%)
Other	40 (3.5%)
No occupation	32 (1.6%)
Decision-making (*n* = 1070)	
No	517 (44.4%)
Yes	553 (55.6%)

### Antenatal care (ANC) service utilization

The ANC service utilization among PVTGs was considerably higher than national average for 10 of 12 indicators; for example, pregnancy registration (PVTGs 99.6% versus national 93.8%), MCP card received (PVTGs 99.6% versus national 90%), early ANC (71.9% versus 70%), four ANC visits (66.3% versus 58.5%), two or more tetanus toxoid (TT) recieved (87.7% versus 83.1%), iron folic acid (IFA) received (98.8% versus 87.6%), 100 or more IFA consumed (54.1% versus 44.1%), long-lasting insecticidal net (LLIN) usage (67.5% versus 39%), supplementary nutrition received (97.7% versus 69%) and ANC counselling (82.7% versus 50.6%) are the ones in which the PVTGs significantly outperformed the average Indian woman in service utilization. However, they lagged behind the national average in two ANC indicators—early pregnancy registration (PVTGs 67% versus national 79.9%) and five ANC components (80.8% versus 82.3%; Table 3).

**Table 3:** Weighted utilization rates of MNCH services by mothers (who gave birth in last 5 years) and child aged 12–23 months

Indicators	PVTGs (*n* = 1186)	Odisha (*n* = 7144)	National (*n* = 176 843)
	Unweighted *n* (weighted %)		
Antenatal care (ANC) services			
Pregnancy registration	1180 (99.6%)	7037 (98.3%)	165 941 (93.8%)
Early pregnancy registration	780 (67%)	6281 (86.0%)	141 712 (79.9%)
MCP card received	1169 (99.6%)	7004 (97.7%)	159 518 (90.0%)
Early ANC	781 (71.9%)	5571 (76.9%)	123 817 (70.0%)
Four ANC visits	687 (66.3%)	5715 (78.1%)	101 435 (58.5%)
Five ANC components	867 (80.8%)	6677 (92.8%)	145 544 (82.3%)
Two or more TT	1014 (87.7%)	6484 (90.8%)	145 310 (83.1%)
IFA received	1164 (98.8%)	6968 (97.2%)	154 235 (87.6%)
> = 100 IFA consumed	629 (54.1%)	4441 (60.8%)	75 254 (44.1%)
LLIN usage	762 (67.5%)	5819 (77.9%)	70 718 (39.0%)
Received supplementary nutrition	1134 (97.7%)	6870 (95.5%)	123 335 (69.0%)
ANC counselling	866 (82.7%)	5815 (78.3%)	89 951 (50.6%)
Intranatal care (INC) services			
Institutional delivery	863 (77.9%)	6583 (93.1%)	155 232 (90.1%)
SBA-assisted delivery	926 (80.4%)	6589 (92.4)	157 114 (90.6%)
Public ambulance service	658 (72.2%)	3187 (42.6%)	50 536 (28.5%)
Postnatal care (PNC) services			
Timely PNC	1156 (98.5%)	6352 (88.5%)	131 589 (76.1%)
Baby weighed	1046 (90.3%)	7016 (98.3%)	161 596 (92.2%)
Early initiation of breastfeeding	711 (60.5%)	4890 (66.9%)	75 645 (41.7%)
Immunization (12–23 months)	*n* = 265	*n* = 1534	*n* = 40 687
All basic vaccination	224 (86.0%)	1410 (90.5%)	31 223 (76.8%)

In comparison with Odisha state averages, PVTG mothers showed lower utilization of ANC services. However, for the indicators such as pregnancy registration (PVTG 99.6% versus Odisha 98.3%), MCP card received (PVTGs 99.6% versus Odisha 97.7%) and IFA received (97.2% versus 98.8%), received supplementary nutrition (PVTGs 97.7% versus Odisha 95.5%) and ANC counselling (PVTGs 82.7% versus Odisha 78.3%), PVTG mothers recorded higher utilization of ANC services (Table 3).

### Intranatal care (INC) service utilization

The institutional delivery rate among PVTGs was 77.9%, and SBA-assisted delivery rate was 80.4%. The corresponding rates were higher for Odisha and India. However, public ambulance service utilization rates were higher (72.2%) among PVTG mothers as compared with their counterparts (Odisha 42.6%, India 28.5%; Table 3).

### Postnatal care (PNC) service utilization

In comparison to national average, Odisha PVTGs recorded higher utilization of timely PNC of mother and newborn (both for home and institutional deliveries; PVTGs 98.5% versus national 76.1%) and early initiation of breastfeeding (60.5% versus 41.7%). However, one of the PNC indicators, that is, newborn weighed, was slightly less than national average (90.3% versus 92.2%).

Moreover, when Odisha PVTG estimates were compared with Odisha state averages, all other indicators like newborn weighed (PVTG 90.3% versus Odisha 98.3%) and early initiation of breastfeeding (PVTG 60.5% versus Odisha 66.9%) were slightly on the lower side except timely PNC of mother and newborn (PVTG 98.5% versus Odisha 88.5%; Table 3).

### Immunization

The proportion of children aged 12–23 months receiving all basic vaccinations was 86%, which is slightly less than the Odisha average, 90.5%, and these rates were substantially higher than national immunization rate (76.8%; Table 3).

### Place of delivery, immunization and caesarean section

**Fig. 1:**
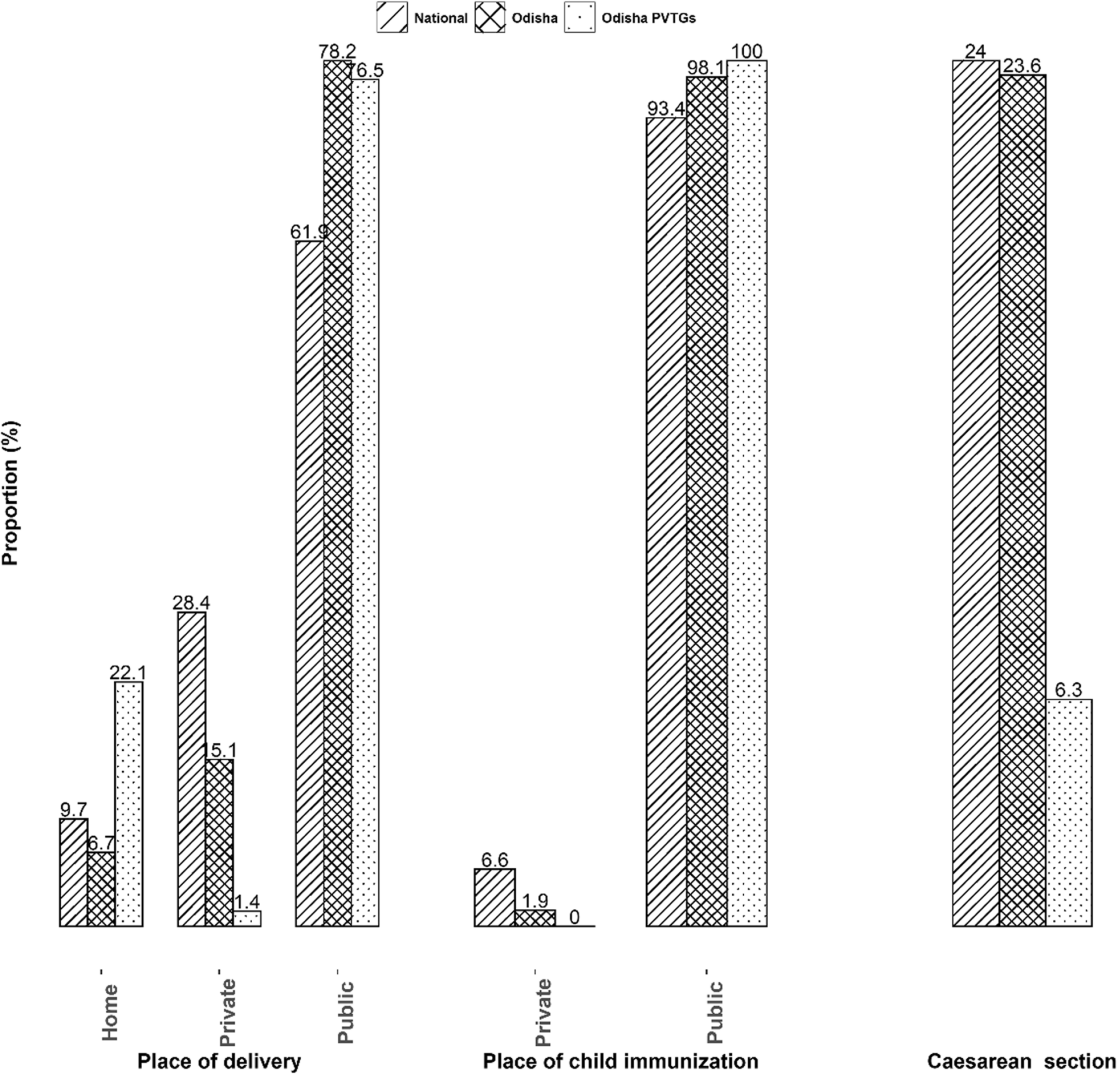
Weighted proportion (%) describing places of delivery, place of child immunization and caesarean section

The majority of the PVTG mothers preferred the public healthcare sector for their institutional delivery (PVTG public sector delivery 76.5%). This rate was higher than that of the nation and almost on par with the Odisha state average (national 61.9% versus Odisha 78.2%). Moreover, every U5 PVTG child received their immunization through the public sector, which is higher than the rate of the nation (93.4%) and the state (98.1%). Nevertheless, the rate of caesarean section (C-section) was less (6.3%) among the PVTGs than the national (24%) and the state (23.6%) rates (Fig. 1).

**Fig. 2:**
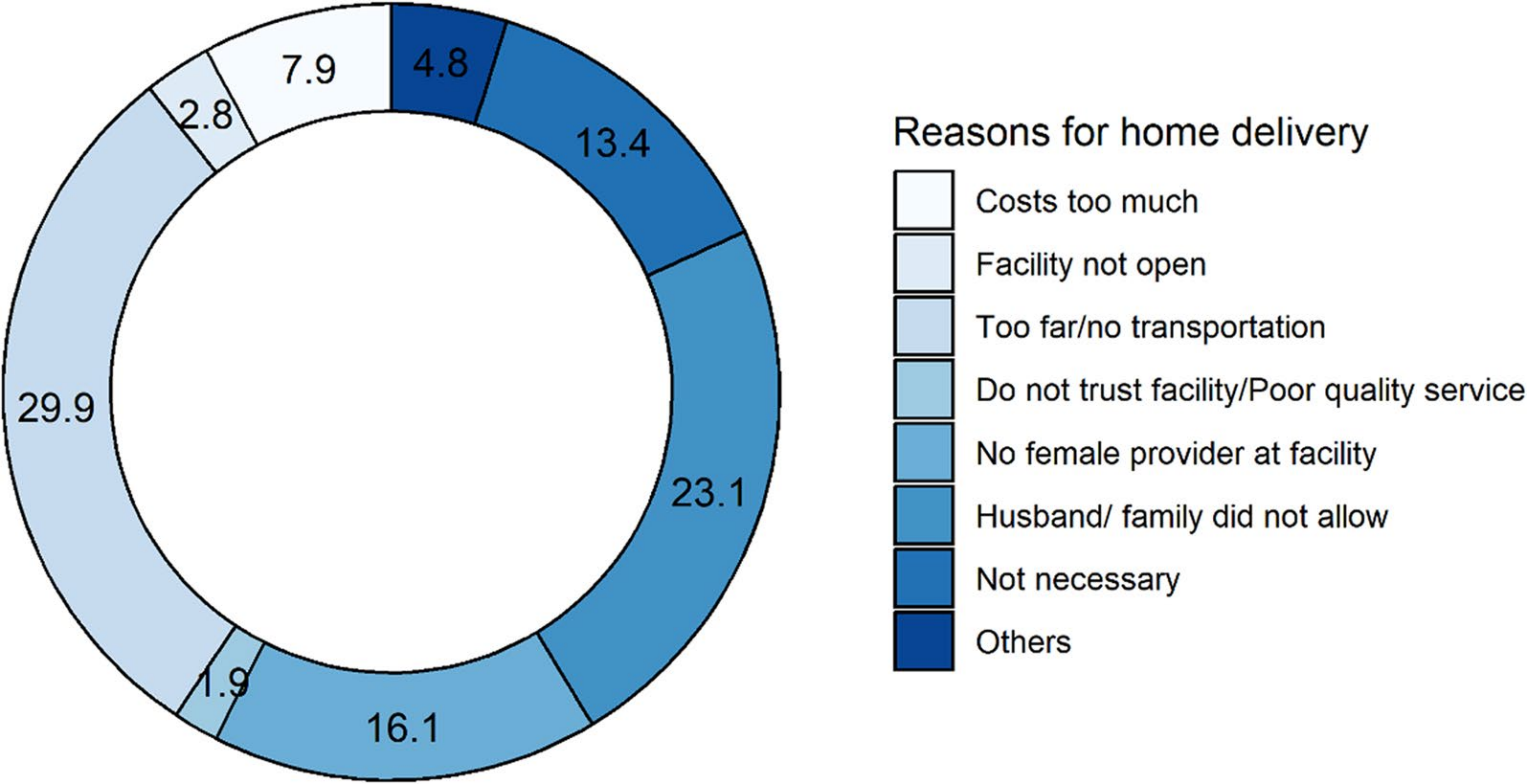
Illustration of reasons behind not opting for institutional delivery [weighted proportion (%)]

For the PVTG mothers who delivered at home, the most common reason that surfaced was that the facility was too far or that there was a lack of transportation (29.9%) followed by the husband and or family not allowing it (23.1%; Fig. 2).

## Discussion

This is the first study to the best of our knowledge that explores the maternal newborn and child health (MNCH) service utilization by particularly vulnerable tribal groups (PVTGs) of India. This study was conducted on a probability representative sample of all the 13 PVTGs residing in the eastern Indian state of Odisha. And to put the service utilization of these key populations in the right perspective, we also compared their MNCH indicators with that of national and Odisha averages.

Overall, the utilization rate of the PVTGs of Odisha was on par with or better than the national averages, barring few domains, and not very different from the utilization rates of the state. This is a remarkable finding and is being reported for the first time from a sizeable sample of this special subpopulation, which is often considered as the most underreached, underserved, marginalized, isolated and vulnerable subpopulation of the country.

The most utilized ANC services among PVTGs were pregnancy registration, MCP card receipt, early ANC, four ANC visits, two or more TT and IFA received, 100 or more IFA consumed, LLIN usage, supplementary nutrition receipt, and ANC counselling. Here, PVTGs significantly outperformed the average Indian woman in ANC service utilization. Odisha is one of the high-focus states of India, also known as empowered action group (EAG) states, a group of states considered lagging in different dimensions of development [23]. But EAG states are also referred to as “aspirational”, as they can make serious strides and that too promptly, to improve their developmental indicators. Odisha seems to have lived up to this “aspirational” tag literally, at least in covering its pregnant women and their children with MNCH services. It demonstrated considerably higher MNCH service utilization rates than the national averages. “A high tide lifts all boats” the saying goes, even the smallest boats. That is exactly what seems to have happened with achievement of high utilization rates by Odisha, which have “lifted” the rates of its most vulnerable subpopulation on par with and often above the national averages. There are PVTG-focused bottom–up approach programmes such as Odisha PVTG Empowerment and Livelihoods Improvement Programme (OPELIP) that are being implemented in the state since 2015 [24]. It is believed that the state has been able to reach the “last habitation” of the PVTGs through the programme. There are other such programmes targeted at PVTGs in the state such as those targeting their nutritional development [24]. Such programmes illustrate the political will of the state to reach out to and serve this key vulnerable population holistically. All of these efforts must have translated into the impressive MNCH utilization rates by the PVTGs of Odisha.

However, the rate of early pregnancy registration among PVTGs was 13 points less than the national average. This is perhaps due to lack of education and awareness among the PVTG mothers regarding signs of pregnancy, reproductive health and the importance of registration and subsequent ANC. This probably leads to delay in the passive reporting to the health system by pregnant PVTG women until they are in a slightly more advanced stage of pregnancy. It can be argued that their geographical remoteness and restricted access to the health system due to other reasons such as social isolation may be underlying this delay. But higher utilization rates in almost all other services perhaps negates this “remoteness” theory; had there been a serious access barrier due to distance, other indicators would have been on the lower side too. Education was found to be a bigger risk factor for registration delay in similar setting in sub-Saharan Africa [25]. So, we also assume that this is due to lack of sexual- and reproductive-health-related education among adolescent girls that is driving the delay among PVTGs. But it is also apparent from our study that, notwithstanding the delay, once the would-be PVTG mothers came in contact with the health system through the registration process, the system sprang into action, resulting in enhanced uptake of subsequent services by the PVTG women, almost akin to that of their non-PVTG counterparts of the state.

Another domain where PVTGs lagged behind the Odisha and national average was institutional delivery rate (by 15.2 and 12.2 points, respectively). Our study probed further into the causes of home delivery and found that geographical remoteness and cultural taboos around hospital admissions, followed by shortage of healthcare providers and paramedics (with reference to Indian Public Health Standards), were the most prominent reasons. Further investigation showed that 8% of the “unintended home deliveries” took place in the vehicle during transit to health facilities, signifying their delay in establishing timely contact with the ambulance services due to lack of telephonic connectivity and delay in transport due to disruption in all-weather-motorable roads during peak of rainy season in the mostly remote places where they live. However, their higher utilization of public ambulance services than the national and state averages again demonstrate the significant reach of the state health system to this key population once contacted.

Despite the geographical remoteness of the PVTGs, perhaps due to the efforts of frontline health workers and due to effective community participation (as observed during the field visits), the PNC service utilization rates of these groups (timely PNC of mother and child, baby weighed and early initiation of breastfeeding) were higher than the national averages. It is worthwhile to note that the utilization of postnatal care by the PVTG mother and their children was on the higher side, as appropriate PNC was received even for home deliveries. Similarly, due to strong implementation of immunization-related outreach services in the state—Mission Indradhanush [26]—the 12–23-month-old PVTG children could achieve immunization rate on par with the state and nation.

The low C-section rate (6.3%) among institutionalized PVTG mothers also demonstrates that medically unindicated elective C-section, which is mostly inappropriate, is rare in this community as opposed to the rest of the nation (one in five women in India undergoes C-section) [27].

The place of delivery and immunization opted for by the PVTGs was entirely the public sector, whereas the private sector had a significant contribution in rest of the country, at least in terms of place of delivery (almost one in four Indian women delivered in a private hospital). This perhaps indicates that the PVTGs rely on the state health system. But it cannot be entirely ruled out that this, to a considerable extent, may be due to their lack of affordability and/or absence of for-profit private options in those remote locations. However, the MNCH utilization indicators of the most marginalized and vulnerable subpopulation of the country being almost on par with or often better than the national average undoubtedly underscores the tremendous equity that has been achieved at the MNCH supply and input side in Odisha. And it is evident that this was achieved mostly through public investment in the proverbial “last mile connectivity” by the welfare state. Whether this equitable utilization is a phenomenon confined to the PVTGs of Odisha only or has spread to other Indian states needs further investigation.

## Limitation and strengths

Our study has few limitations. First, we could not get much literature in this domain to compare our results with other PVTGs of India and similar vulnerable population from other parts of the world because there is dearth of evidence regarding these communities. Second, our study is restricted to the PVTGs of Odisha. There may be sociocultural issues specific to regions and PVTGs which may be influencing their reproductive health decisions. For instance, during field visit it was observed anecdotally that quite a few pregnant women from some PVTGs of Odisha delayed their pregnancy registration on the belief that early disclosure of pregnancy may lead to casting of the evil eye. Therefore, the study results may not be generalized to the other PVTGs residing in other parts of the country. Lastly, our study could not present PVTG-wise stratified estimates due to inadequacy of sample size for each PVTG, which future research should address.

However, the lack of evidence from such very vulnerable communities can actually be considered the strength of our study, because we examined a sizeable sample of PVTG mothers and children for the first time, to the best of our knowledge, large enough for pooled estimates of PVTGs. We also used mostly MNCH indicators that are harmonized with NFHS, which afforded us the comparison of the estimates of the PVTGs with those of Odisha and India.

## Policy implications

Our study showed that the PVTG women have little formal education in comparison with other groups. Lack of education leads to delays in establishing contact with the welfare state or sometimes no establishment of contact at all, which is perhaps reflected in substantial delays in pregnancy registration in this group. For the short term, until their educational endowments can be brought to the level of the rest of the state, efforts should be made to at least informally educate the PVTG adolescent girls, would-be mothers and their parents and parents-in-law about life skills, reproductive- and sexual-health-related issues and the MNCH programme, and most importantly the benefits of prompt utilization of its cascade of services once pregnancy is identified. The same principles may be applied for increasing the rates of institutional delivery among them, which is also on the lower side. Additionally, increasing tele-connectivity to these remote areas where PVTGs live should be a major thrust area for the state because that is often a critical barrier to accessing the health and other welfare services by this community, especially with regards to summoning transports or ambulances while travelling to health facilities.

The federal government of India has recently introduced the Pradhan Mantri PVTG Development Mission for the PVTGs of India. This mission should be leveraged to achieve the structural changes (education, health, nutrition and connectivity) in this subpopulation keeping their identity intact. These changes are likely to further enhance their interaction with the health system and increase their utilization of healthcare services.

## Future road map

It is time to make the most vulnerable visible. Future research should explore whether encouraging utilization rates of MNCH services are also leading to better MNCH outcomes among them. The lack of data on the health of women and adolescent girls of these subpopulation might be disguising the disparities between populations, preventing instantaneous action to address it. Therefore, management information systems of MNCH and also other health programmes should collect/record caste/tribe disaggregated data.

## Conclusion

To conclude, this is the first study exclusively on PVTGs, purportedly the most vulnerable population of India, examining comprehensively their utilization of MNCH services. The study was conducted in Odisha on a representative sample of all the 13 PVTGs of state. It is very pleasantly surprising to note that the PVTGs have achieved comparable or often more utilization rates than the national average. This can be attributed to Odisha state as a whole achieving high utilization rate, thereby dragging upwards the rates of their PVTGs. However, PVTGs have underperformed in two indicators, mainly timely pregnancy registration and institutional delivery, which should be urgently addressed by the state health and other system. As the study was only conducted in PVTGs of Odisha, this scenario may not be generalizable to the entire PVTG population of India, which future research should endeavour to cover.

### Supplementary Information

**Additional file 1: Table S1.** The overall health status in Odisha and scheduled tribes of Odisha, India. **Table S2.** Public health system of Odisha (2021) [18]. **Table S3.** Operational definition of maternal, newborn and child health indicators

## Data Availability

The data generated or analysed during the current study are available from the corresponding author on reasonable request.

## Abbreviations

MNCH: Maternal newborn and child health
MMR: Maternal mortality ratio
OECD: Organization for Economic Cooperation and Development
U5MR: Under 5 years child mortality rates
ST: Scheduled tribes
PVTGs: Particularly vulnerable tribal groups
NFHS: National Family Health Survey
LASI: Longitudinal Ageing Study of India
MPA: Micro-project areas
BDA: Bonda Developmental Agency
DDA: Didayi Developmental Agency
JDA: Juang Developmental Agency
CBDA: Chuktia Bhunjia Developmental Agency
DKDA: Dangaria Kandha Developmental Agency
LSDA: Lanjia Saora Developmental Agency
SDA: Saora Developmental Agency
TDA: Tumba Developmental Agency
KKDA: Kutia Kandha Developmental Agency
PBDA: Paudi Bhuyan Developmental Agency
HK&MDA: Hill-Kharia and Mankirdia Developmental Agency
ANC: Antenatal care
MCPC: Mother and child protection card
INC: Intranatal care
SBA: Skilled birth attendant
PNC: Postnatal care
BCG: Bacillus Calmette-Guerin
TT: Tetanus toxoid
IFA: Iron folic acid
LLIN: Long-lasting insecticidal nets
OPELIP: Odisha PVTG Empowerment and Livelihoods Improvement Programme
