# Supplementary material for: To leave no one behind: Assessing utilization of maternal newborn and child health services by all the 13 particularly vulnerable tribal groups (PVTGs) of Odisha, India
Source: Health Res Policy Syst. 2024 Jan 22;22:12. doi: 10.1186/s12961-023-01101-7 (PMC10802067; doi:10.1186/s12961-023-01101-7)
Supplement: Supplementary file 1 — Additional file 1: Table S1. The overall health status in Odisha and scheduled tribes of Odisha, India. Table S2. Public health system of Odisha (2021) [18]. Table S3. Operational definition of maternal, newborn and child health indicators [file 12961_2023_1101_MOESM1_ESM.docx]

Supplementary Table 1. The overall health status in Odisha and Scheduled tribes of Odisha, India.

| **Indicators** | **Odisha (2019-2021)** | **Scheduled Tribes of Odisha (2015-2016)** |
| --- | --- | --- |
| Total fertility rate (children per women) | 1.8 | 2.46 |
| Neonatal mortality rate (per 1,000 live births) | 27 | 35.5 |
| Infant mortality rate (per 1,000 live births) | 36.3 | 51.8 |
| Under-five mortality rate (per 1,000 live births) | 41.1 | 65.6 |
|  |  |  |

Supplementary Table 2. Public health system of Odisha (2021) (18)

| **Items** | **Required** | **In position** | **Shortfall** |
| --- | --- | --- | --- |
| Sub Center | - | 6688 | - |
| Primary Health Centres (PHCs) | - | 1288 | - |
| Community Health Centres (CHCs) | - | 377 | - |
| Health Worker [Female] / ANM at Sub Center & PHCs | 7976 | 7579 | 397 |
| Allopathic Doctors at PHCs | 1288 | 926 | 362 |
| Total Specialists [Surgeons, OB&GY, Physicians & Paediatricians] at CHCs | 1508 | 309 | 1199 |
| Radiographers at CHCs | 377 | 70 | 307 |
| Pharmacists at PHCs & CHCs | 1665 | 1701 | - |
| Laboratory technicians at PHCs & CHCs | 1665 | 839 | 826 |
| Nursing Staff at PHCs & CHCs | 3927 | 2279 | 1648 |
|  |  |  |  |

Supplementary table 3. Operational definition of maternal, newborn and child health indicators

| **Indicators** | **Denominator** | **Operational definition** |
| --- | --- | --- |
| ***Antenatal care (ANC) services*** | | |
| Pregnancy registration | 1186 | It includes registration of current pregnancy at the nearest health facility as soon as pregnancy is detected |
| Early pregnancy registration | 1180 | Registration of pregnancy within first trimester |
| Received MCP Card | 1180 | Received mother and child protection card after registration of pregnancy |
| Early ANC | 1186 | Received antenatal check-up within first trimester of pregnancy |
| Four ANC visits | 1186 | Received 4 or more antenatal check-up |
| Five ANC components | 1186 | Examination of weight, haemoglobin, blood pressure, urine, and abdomen during antenatal visits |
| Two or more TT | 1186 | Received 2 or more tetanus toxoid injection during pregnancy |
| IFA received | 1185 | Received iron folic acid tablets during pregnancy |
| >=100 IFA consumed | 1158 | Consumed 100 or more iron folic acid tablets during pregnancy |
| LLIN usage | 1186 | Regularly used LLIN during pregnancy |
| Received supplementary nutrition | 1186 | Received ‘take home ration’ during pregnancy |
| ANC counselling | 1186 | Received counselling on topics like institutional delivery, cord care, breastfeeding, keeping baby warm, and family planning |
| ***Intranatal care (INC) services*** | | |
| Institutional delivery | 1186 | Delivery of the baby at private or public institution |
| SBA assisted delivery | 1186 | Delivery assisted by skilled birth attendant |
| Public ambulance service | 970 | Availed public ambulance to reach the healthcare delivery institution |
| ***Postnatal care (PNC) services*** | | |
| Timely PNC | 1186 | Received postnatal care within 48 hours of delivery both by mother and their children (for institutional and home delivery) |
| Baby weighed | 1186 | Weight measurement of the newborn |
| Early initiation of breastfeeding | 1181 | Initiated breastfeeding immediately within one hour of delivery |
| ***Immunization (12-23 months)*** | | |
| All basic vaccination | 265 | Received all-basic vaccines such as Bacillus Calmette-Guerin (BCG), three doses of the polio vaccine, three doses of pentavalent vaccine (Diphtheria, Pertussis, Tetanus (DPT), Hepatitis B and Hib), first dose of measles vaccine for the age group 12-23 months. |
